# The Blood-Brain Barrier Permeability of Six Indole Alkaloids from *Uncariae Ramulus Cum Uncis* in the MDCK-pHaMDR Cell Monolayer Model

**DOI:** 10.3390/molecules22111944

**Published:** 2017-11-10

**Authors:** Yi-Nan Zhang, Yan-Fang Yang, Wei Xu, Xiu-Wei Yang

**Affiliations:** State Key Laboratory of Natural and Biomimetic Drugs, Department of Natural Medicines, School of Pharmaceutical Sciences, Peking University Health Science Center, Peking University, No. 38, Xueyuan Road, Haidian District, Beijing 100191, China; zhangyn1994@126.com (Y.-N.Z.); yangyanfang@bjmu.edu.cn (Y.-F.Y.); high-xu@163.com (W.X.)

**Keywords:** *Uncariae Ramulus Cum Uncis*, Rubiaceae, Indole alkaloids, MDCK-pHaMDR cell monolayer model, Blood-brain barrier, Permeability

## Abstract

*Uncariae Ramulus Cum Uncis* (URCU) is a widely used traditional Chinese medicine, and is reported to have various central nervous system effects. Alkaloids have been demonstrated to be the predominant pharmacological active components of URCU. In order to evaluate the blood-brain barrier (BBB) permeability and transport mechanism of six typical indole alkaloids from URCU, the MDCK-pHaMDR cell monolayer model was used as an in vitro surrogate model for BBB. The samples were analyzed by high-performance liquid chromatography, and the apparent permeability coefficients (*P*_app_) were calculated. Among the six alkaloids, isorhynchophylline (**2**), isocorynoxeine (**4**), hirsutine (**5**) and hirsuteine (**6**) showed high permeability, with *P*_app_ values at 10^−5^ cm/s level in bidirectional transport. For rhynchophylline (**1**) and corynoxeine (**3**), they showed moderate permeability, with *P*_app_ values from the apical (AP) side to the basolateral (BL) side at 10^−6^ cm/s level and efflux ratio (*P*_app BL→AP_/*P*_app AP→BL_) above 2. The time- and concentration-dependency experiments indicated that the main mechanism for **2**, **4**, **5** and **6** through BBB was passive diffusion. The efflux mechanism involved in the transports of compounds **1** and **3** could be reduced significantly by verapamil, and molecular docking screening also showed that **1** and **3** had strong bindings to *P*-glycoprotein. This study provides useful information for predicting the BBB permeability for **1**–**6**, as well as better understanding of their central nervous system pharmacological activities.

## 1. Introduction

**Figure 1:**
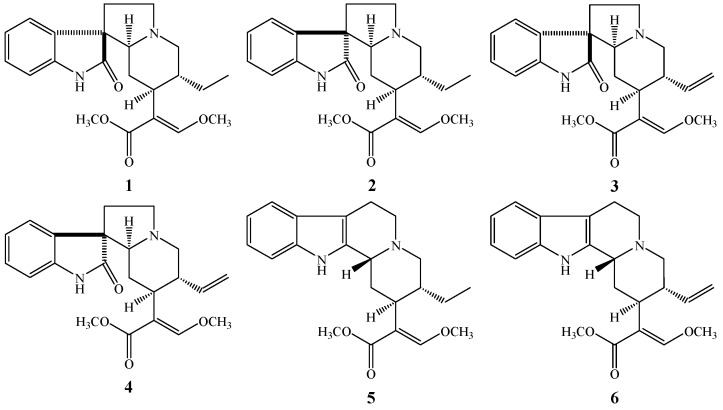
Chemical structures of rhynchophylline (**1**), isorhynchophylline (**2**), corynoxeine (**3**), isocorynoxeine (**4**), hirsutine (**5**) and hirsuteine (**6**).

*Uncariae Ramulus Cum Uncis* (URCU), the dried hook-bearing branch of *Uncaria rhynchophylla* (Miq.) Miq. ex Havil., *U. macrophylla* Wall., *U. hirsuta* Havil., *U. sinensis* (Oliv.) Havil. or *U. sessilifructus* Roxb. (family Rubiaceae) [1], has been used as a traditional Chinese medicine (TCM)—called Gou-Teng, in Chinese—for thousands of years. In the clinic, URCU has been prescribed in many formulas to treat hypertension, headache, epilepsy, senile dementia and eye diseases [2]. Alkaloids are the predominant pharmacologically active components of URCU, and more than 120 alkaloids have been isolated and identified from it [3]. Rhynchophylline (**1**), isorhynchophylline (**2**), corynoxeine (**3**), isocorynoxeine (**4**), hirsutine (**5**), and hirsuteine (**6**) (Figure 1) are representative indole alkaloids from URCU. It has been reported that alkaloids **1**–**5** exhibit inhibitory activity on lipopolysaccharide-induced NO release on microglial cells [4,5], alkaloids **1**, **2**, **4**–**6** could protect against glutamate-induced cell death on PC12 cells or cultured cerebellar granule cells [6,7], and that alkaloid **2** could attenuate the Aβ_25–35_-induced neuronal apoptosis in hippocampus and ameliorate cognitive deficits [8]. Pharmacokinetic studies have shown that alkaloids **1**–**6** were able to be detected in plasma after oral administration in rats [9,10,11,12,13]. However, the blood-brain barrier (BBB) permeability and transport mechanism of the six alkaloids have been rarely studied. Therefore, it is necessary to study the penetration abilities of the alkaloids through the BBB for accessing their central nervous system (CNS) pharmacological activities.

The BBB, formed by the endothelial cells lining microvessels, separates the blood from the brain interstitial fluid and provides homeostasis for complex neural function, as well as protecting the CNS from circulating toxins [14]. Therefore, the BBB is considered to be the most important barrier for drugs with CNS activities, and the CNS activities for different drugs and drug formulations may be highly relevant in terms of their in vivo BBB permeation level [15]. To predict the permeability of drug candidates, several cell models have been developed to mimic the BBB, such as cEND cells [16] and endothelial-astrocytes coculture cells [17]. Among these in vitro cell models, the MDCK-MDR cell monolayer model has been evaluated as a convenient permeability screen for BBB [18,19,20]. The MDCK-pHaMDR cell used in this study is derived from the parental MDCK cell line after infection with the MDR1 virus, with similarities to BBB in terms of its morphology and high expression of *P*-glycoprotein (*P*-gp) [21]. It has been used to investigate the BBB permeability of components in TCM, such as coumarins from Angelicae Pubescentis Radix [22] and lignans from the seeds of *Myristica fragrans* [23]. Therefore, the MDCK-pHaMDR cell monolayer was used as a reliable in vitro model to investigate the BBB permeability of six alkaloids from URCU in this study.

In this paper, the in vitro BBB transport of six alkaloids from URCU was examined in MDCK-pHaMDR cell monolayer model to predict their BBB permeability. Their interactions with *P*-gp were also carried out by in vitro verapamil inhibition and in silico molecular docking. The aim of the study was to discover the substance basis for the CNS activities of URCU.

## 2. Results and Discussion

### 2.1. Validation of the MDCK-pHaMDR Cell Monolayer

The integrity of differentiated MDCK-pHaMDR cell monolayers was validated before transport experiments by measuring the transepithelial electrical resistance (TEER) with an epithelial volt-ohm meter (EVOM; World Precision Instruments). Only cell monolayers with a TEER value above 1000 Ω·cm^2^ were used for further study [24]. The apparent permeability coefficient (*P*_app_) values of caffeine and atenolol, well- and poorly transported markers by passive diffusion across BBB, were calculated as (3.00 ± 0.11) × 10^−5^ cm/s and (2.87 ± 0.15) × 10^−7^ cm/s, respectively. The results were in good agreement with the reported values [18], verifying the applicability of the cell monolayer as an in vitro BBB model.

Rhodamine 123 (Rh123), a typical *P*-gp substrate, was used as a positive control for the function of *P*-gp on MDCK-pHaMDR cell monolayers [18]. The efflux mechanism was apparent in its transport through the cell monolayer. When co-incubated with verapamil (a classical *P*-gp inhibitor), the efflux of Rh123 was inhibited, and the efflux ratio decreased from 6.88 (in the absence of verapamil) to 1.34 (in the presence of verapamil). This result indicated that *P*-gp was stably expressed in the cell model, and could have an obvious effect on drug efflux transport (Table 1).

**Figure 2:**
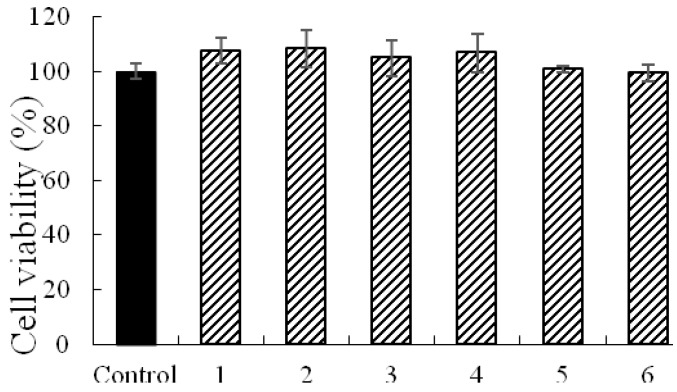
The cell viability of the six alkaloids at 100 μM on MDCK-pHaMDR cells for 6 h.

Cell viability assays showed that alkaloids **1**–**6** at the maximum test concentration of 100 µM exerted no significant influence on cell viability (Figure 2).

### 2.2. Validation of High-Performance Liquid Chromatography Analysis Method

The high-performance liquid chromatography (HPLC) methods for alkaloids **1**–**6** were validated. The standard calibration curves were constructed by plotting peak area (*y*) vs. concentration (*x*, µM). Regression equations, coefficient correlations (*r*^2^) and concentration ranges of the calibration curves for the six alkaloids are shown in Table 2. The methodological evaluation showed that intraday and interday precision was less than 4.63% and 12.43%, respectively, while accuracy was between 99.37% and 113.34%. The six alkaloids were stable, with relative standard deviations (RSD) ranging from 96.32% to 108.52% after three freeze-thaw cycles. The data are summarized in Table 3, and the HPLC methods were validated to be in compliance with the Guidance for Industry Bioanalytical Method Validation of the FDA [25].

### 2.3. Bidirectional Transport Determination

#### 2.3.1. Bidirectional Transport of Alkaloids **1**–**6**

The bidirectional *P*_app_ values and the efflux ratios for alkaloids **1**–**6** are summarized in Table 4. The bidirectional *P*_app_ values of **2**, **4**, **5**, **6** were at a level of 10^−5^ cm/s, which is similar to that of caffeine; therefore, they were classified as well-absorbed compounds through BBB. Their efflux ratio values were less than 2, suggesting that there was no significant efflux mechanism involved. As for alkaloids **1** and **3**, the efflux ratio was much more than 2, indicating that carrier-mediated efflux may be involved in transport. Therefore, verapamil, a classical *P*-gp inhibitor, was further used to verify their absorption pathway.

#### 2.3.2. Intracellular Accumulation and Recovery

The intracellular accumulation (the amounts of the compounds accumulating in cells and binding to cells) and recovery (the total amounts of the compounds on both sides of the insert and intracellular) of the six alkaloids were also measured to check the mass balance. As shown in Table 5, the intracellular accumulation rates of **1**–**4** were very low (less than 0.4%). By contrast, the intracellular accumulation rates of **5** and **6** were much higher, more than 16% in the AP→BL direction and 6% in BL→AP direction. These results showed that **5** and **6** were more likely to accumulate in cells, which might be because their Log D at pH 7.35 (Table 4) was higher, so they were more lipophilic than **1**–**4**. The total recovery rates of all test compounds were high (above 88%), indicating that they were stable and hardly metabolized in MDCK-pHaMDR cells.

#### 2.3.3. The Time Course and Concentration-Dependence of Permeation of Alkaloids **1**–**6**

**Figure 3:**
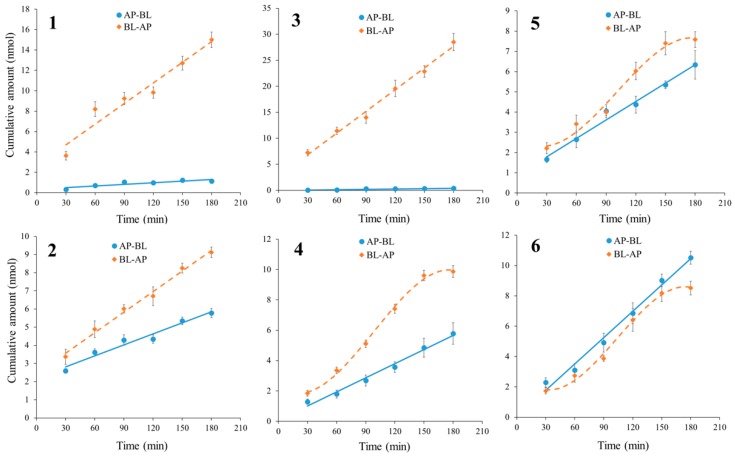
The bidirectional transport cumulative amounts of six alkaloids in MDCK-pHaMDR cell monolayer as a function of time from 30–180 min. Alkaloids **1**–**6** were at 40 μM. Data are the mean ± SD (*n* = 6).

In the bidirectional time-dependency experiments, the cumulative amounts of alkaloids **1**–**6** increased almost linearly with time in both directions (Figure 3). The curves for alkaloids **4**, **5** and **6** in BL→AP showed plateau periods after 150 min, a possible explanation for which is the concentration differences between the receiver and donor sides of the test compounds reaching a balance such that transport progress would slow down. For alkaloids **1** and **3**, it was obvious that the transport amount in BL→AP accumulated much faster than that of AP→BL, which wasmainly caused by their carrier-mediated efflux transport mechanism, as discussed above.

**Figure 4:**
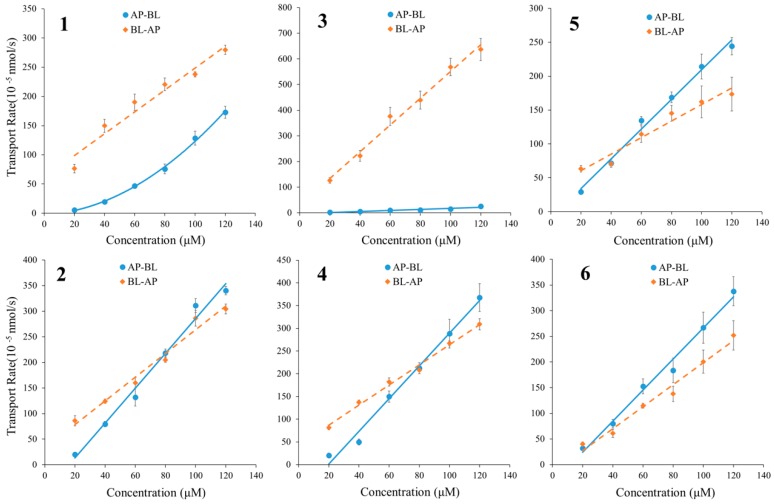
The bidirectional transport rates of six alkaloids in MDCK-pHaMDR cell monolayer as a function of concentration. Alkaloids **1**–**6** were at 20–120 μM for 90 min. Data are the mean ± SD (*n* = 6).

Meanwhile, the transport rates of alkaloids **2**, **4**, **5** and **6** increased nearly linearly with the increase of their concentration, and were similar in AP→BL and BL→AP directions (Figure 4). Therefore, passive diffusion related to the concentration difference between two sides was able to be verified in the transport of alkaloids **2**, **4**, **5** and **6**. As to **1** and **3**, the transport rate in the BL→AP direction was significantly higher than that of AP→BL, indicating a rapid efflux during the passive transport.

#### 2.3.4. The Relationship between Permeability and Drug Properties

The physicochemical properties of different compounds might affect their BBB permeabilities. The relationship between the BBB permeability and log D of coumarins and lignans has been verified in our previous work [22,23]. In this study, however, the analysis results according to the previous method were not satisfied, probably because there were two pairs of isomers (**1**–**2**, **3**–**4**) with the same MW and logD.

Another common measure of the degree of BBB penetration is the ratio of the steady-state concentrations of the drug molecule in the brain and in the blood, usually expressed as log(C_brain_/C_blood_) or, more simply, log BB [26]. The log BB values of the six analytes were calculated from the modified Clark’s equation [16,26] as follows:

log BB = 0.152logP − 0.0148PSA + 0.139

The calculated log BB values of the six alkaloids, along with the log P and PSA values (calculated by Pallas 3.3.2.6 ADEM/Tox Software.), are listed in Table 6. Compounds with log BB values of >0.3 were readily distributed to the brain, whereas compounds with log BB values of <−1.0 were poorly distributed to the brain [26]. As the log BB values of the six alkaloids were between −0.60–−0.18, they were included as moderately- or well-absorbed compounds across BBB. The predicted results coincided with the determined *P*_app_ values in Table 4.

### 2.4. The Interactions between Alkaloids and P-gp

#### 2.4.1. Verapamil Inhibition on the Efflux of Alkaloids **1** and **3**

To verify the efflux and the transporter during the transport of **1** and **3** through BBB, the influence of verapamil on the transport was examined, and the results summarized in Table 4. When transported with 100 µM of verapamil, their *P*_app AP→BL_ increased, while *P*_app BL→AP_ decreased markedly and the efflux ratio reduced to less than 2. The results revealed that **1** and **3** were substrates of *P*-gp, and the *P*-gp-related efflux mechanism was involved in their transport through BBB. The only difference between **1** and **2**, **3** and **4** in chemical structure is C-7 stereochemistry. **1** and **3** are 7(*R*) epimer and **2** and **4** are 7(*S*) epimer. The comparison of **1** and **2**, **3** and **4** indicated that C-7 epimerization significantly affects their binding with *P*-gp.

#### 2.4.2. Molecular Docking of **1**–**6** to *P*-gp

**Figure 5:**
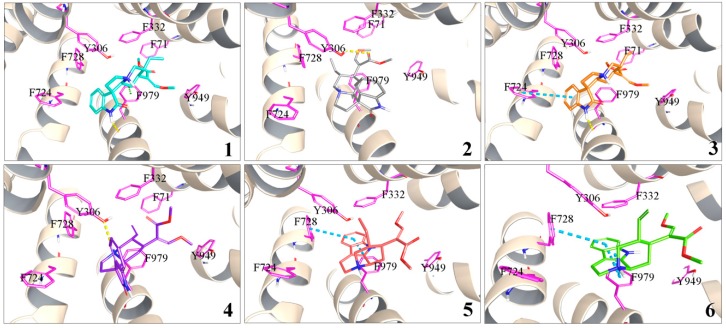
Docked complex of *P*-gp and alkaloids **1**–**6**. Yellow, blue, and green dashed lines represent hydrogen bonds, π-π bond and p-π bond, respectively.

In silico molecular docking has been practically applied in the study of protein-ligand interactions [27]. A mouse *P*-gp crystal structure was used as a receptor to perform molecular docking with alkaloids **1**–**6**, since it has been identified and reported to have 87% of its sequence identical to human *P*-gp [28]. The docked ligand poses and the ligand-receptor interactions were visualized as Figure 5. The GlideScore (GScore) is the index of the binding energy, and a smaller GScore means a stronger binding [29].

Alkaloid **3** had a hydrogen bond with residue Phe 979 and a parallel π-π stacking with Phe 724, while alkaloid **1** had a hydrogen bond and a p-π stacking with Phe 979. As for alkaloids **2** and **4**, there was only a hydrogen bond with residue Tyr 306, so their bindings with *P*-gp were less than for alkaloids **1** and **3**. The GScore of alkaloids **3** and **1** (−8.290 and −7.750 kcal/mol, respectively) also indicated that they had stronger bindings than alkaloids **2** and **4** (−7.264 and −7.169 kcal/mol, respectively). C-7 epimerization might lead to the different binding degree of two pairs of isomers (**1**–**2**, **3**–**4**) in the drug-binding pocket. Alkaloids **5** and **6** were observed to have the largest GScores of −4.621 kcal/mol and −4.383 kcal/mol, respectively, and their interaction with *P*-gp was also weaker, involving only π-π stackings.

The docked complex conformation and interactions suggested that alkaloids **1** and **3** had a stronger binding affinity to *P*-gp than other alkaloids. The results were consistent with the in vitro transport experiment data mentioned above.

## 3. Experimental Section

### 3.1. Assayed Alkaloids

Alkaloids rhynchophylline (**1**), isorhynchophylline (**2**), corynoxeine (**3**), isocorynoxeine (**4**), hirsutine (**5**), and hirsuteine (**6**) were purchased from Push Bio-technology Co. (Chengdu, China); their purities were above 98%, as measured by HPLC with diode array detector (DAD) analysis.

### 3.2. Chemicals and Reagents

Colchicine, caffeine, atenolol, Rh123, verapamil, dimethylsulfoxide (DMSO) and 3-(4,5-Dimethyl-2-thiazolyl)-2,5-diphenyl-2*H*-tetrazolium bromide (MTT) were obtained from Sigma-Aldrich (St. Louis, MO, USA). Dulbecco’s modified Eagle’s medium (DMEM), fetal bovine serum (FBS), 0.25% trypsin-EDTA, penicillin and streptomycin, phosphate buffered saline (PBS), and other culture supplements were purchased from Gibco^®^ Laboratories (Life Science Technologies, Inc., Grand Island, NY, USA). Reagents for Hank’s Balanced Salts Solution (HBSS) and other chemicals were of analytical grade from Beijing Chemical Works (Beijing, China). Methanol (MeOH), acetonitrile (ACN), triethylamine and acetic acid were of HPLC grade (J. T. Backer, Center Valley, PA, USA). 12-Well Transwell^®^ plates with polycarbonate inserts and cell culture flasks were purchased from Corning Inc. (Cambridge, MA, USA). Milli-Q water (Millipore, Bedford, MA, USA) was used throughout the study.

### 3.3. HPLC Analysis

HPLC experiments were performed with a Dionex Ultimate™ 3000 UHPLC system (Dionex Corp., Sunnyvale, CA, USA) comprised of Ultimate 3000 pump, autosampler, column compartment and DAD. The signals were acquired and processed applying a Chromeleon version 6.80 software (Dionex Corp., Sunnyvale, CA, USA). Chromatographic separation was performed on an analytical Dikma Diamonsil^TM^ C_18_ column (250 mm × 4.6 mm, 5 μm, Dikma Technology, Inc., Beijing, China). The flow rate was 1.0 mL/min and the column temperature was 25 °C. The isocratic mobile phase was composed of MeOH–H_2_O (containing 0.1% of triethylamine and adjusted with acetic acid to pH 5.4, *v*/*v*) in 70:30 for alkaloids **1**–**6**. The UV detection was at 245 nm for **1**–**4** and 221 nm for **5** and **6**. Elution peaks were monitored and the peak areas were used to calculate the compound concentrations.

### 3.4. HPLC Method Validation

The stock solutions of the six alkaloids were diluted to three concentrations (low, medium and high) as quality control (QC) samples. Accuracy, precision and stability of QC samples were validated. Accuracy and intraday precision were evaluated by determining the QC samples in three replicates on the same day, while interday precision was assessed by repeated analysis of the same QC samples over three consecutive days. Precision was estimated by RSD, which should not exceed 15%. Accuracy was assessed by comparison of the calculated mean concentrations with the spiked concentrations, and the acceptable range should be within 85–115%. The absolute recoveries of six alkaloids were determined by comparing the peak areas of analytes in QC samples with those of the standard solutions in MeOH with equivalent concentrations. Stability was evaluated by measuring the QC samples after three freeze (–20°C)−thaw (room temperature) cycles, and comparing the results with those of freshly prepared QC samples. The stability was expressed as the mean of percentage remains, and the acceptable range was 85–115%.

### 3.5. Culture of MDCK-pHaMDR Cells

The MDCK-pHaMDR cell line was a gift from Dr. Michael M. Gottesman (National Institute of Health, Bethesda, MD, USA). The cells were cultured at 37°C in 5% CO_2_ using DMEM with d-glucose (4.5 g/L), NaHCO_3_ (3.7 g/L) and sodium pyruvate (110 mg/L), supplemented with 10% FBS, 80 ng/mL of colchicine, 50 units/mL of penicillin and 50 µg/mL of streptomycin [30]. They were seeded onto a 12-well Transwell insert filter at a density of 1.0 × 10^5^ cells/mL. After growing for 8 days, the cells reached confluence and full differentiation for transport studies. The cells in this study were between passages 15 and 25.

### 3.6. Transport Experiments on MDCK-pHaMDR Cell Monolayer

The stock solutions of the six alkaloids, caffeine, atenolol and verapamil were prepared with DMSO. They were further diluted to a designed concentration by HBSS before the experiments. The cytotoxicity of the alkaloids on the MDCK-pHaMDR cells was determined by the MTT assay. MDCK-pHaMDR cells were cultured with alkaloids **1**–**6** at the maximum test concentration of 100 µM for 6 h and the optical density values at 490 nm were read on a Thermo Multiskan MK3 Automated Microplate Reader (Thermo-Labsystems, Franklin, MA, USA).

#### 3.6.1. Bidirectional Transport Experiments of Alkaloids **1**–**6**

The cell monolayers were washed twice with prewarmed HBSS before the experiments were carried out. The HBSS medium in the AP side (0.5 mL, for absorption transport) or BL side (1.5 mL, for efflux transport) was replaced with different test compounds. The plates were shaken at 55 rpm for 90 min at 37 °C in a water bath. Samples were quantitatively collected from both sides of the cell monolayer, immediately frozen, lyophilized, and then preserved below −20 °C. To measure the intracellular amounts, the cell monolayers were lyophilized after three freeze (−20 °C)–thaw (room temperature) cycles and preserved below –20 °C. The integrity and transportation ability of the monolayer were examined by the transports of caffeine and atenolol with the method described above. The tested concentrations of alkaloids **1**–**6** were 40 µM. The function of P-gp were tested by the transport of Rh123 at 10 µM in the presence and absence of verapamil (100 µM) undertaken as described above.

#### 3.6.2. Time- and Concentration-Dependent Transport Experiments of Alkaloids **1**–**6**

In the time-dependent transport experiments, test alkaloids with above-mentioned concentrations were added to either AP or BL side of the inserts for absorption transport (AP→BL) or efflux transport (BL→AP), and incubated for 30, 60, 90, 120, 150 and 180 min. To observe the concentration-dependence, 20, 40, 60, 80, 100, 120 µM of alkaloids **1**–**6** were added to either AP or BL side and incubated for 90 min.

#### 3.6.3. Verapamil Inhibition of Transport

The verapamil inhibition experiment was carried out for alkaloids **1** and **3**. The cells were pre-incubated with 100 µM of verapamil for 30 min before drug transport assays. Then verapamil was added to both sides, and 40 µM of test alkaloid was added to AP or BL side.

#### 3.6.4. Sample Preparations

The lyophilized samples were dissolved in MeOH of proper volume, thoroughly vortex-mixed, ultrasonic for 20 min and then centrifuged at 15,000 × g for 10 min. An aliquot of 20 μL supernatant solution was used for HPLC assay. The lyophilized cell monolayers were ultrasonically extracted with 100 μL of MeOH and then treated as above.

#### 3.6.5. Data Analysis

*P*_app_ in AP→BL or BL→AP direction of each alkaloid was calculated based on the following equation:

*P*_app_ = dQ/dt/A/C_0_ (cm/s)

where Q is the accumulation quantity of the alkaloid in the receiver side (μmol), dQ/dt is the linear appearance rate of the alkaloid in the receiver side (μmol/s), C_0_ is the initial concentration in the donor side (µM), and A is the membrane surface area (cm^2^). All of the experiments were conducted in six duplications and data were expressed as the mean ± SD.

### 3.7. Statistical Analysis

The data were presented as the mean ± SD. Statistical differences were assessed by Student′s *t*-test with SPSS statistics package v.20.0 (SPSS Inc., Chicago, IL, USA).

### 3.8. Molecular Docking

The three-dimensional crystal structure of mouse *P*-gp (PDB code: 4M1M, resolution 3.8Å) was retrieved from RCBS PDB database. The structure was optimized with the Protein Preparation Wizard in Schrödinger Maestro Suite 2017. Any inconsistencies in the structure such as missing hydrogens, incorrect bond orders, orientation of the different functional groups of the amino acids were corrected during the optimization process. The structures of the six alkaloids from URCU were prepared using the LigPrep application in Schrödinger Maestro Suite 2017. LigPrep was used to perform the conversion of structures from two-dimensional to three-dimensional, correction of improper bond distances, bond orders, generation of ionization states and energy minimization processes. The ligands were then docked into *P*-gp using the Induced Fit Docking application of Schrödinger Maestro Suite 2017 with Glide XP (extra precision) mode. The poses are ranked using GScore (kcal/mol), which is calculated from the following equation:

GScore = 0.065vdW + 0.0130Coul + Lipo + Hbond + Metal + BuryP + RotB + Site

where vdW is Van der Waals energy, Coul is Coulomb energy, Lipo is Lipophilic term, Hbond is Hydrogen-bonding, Metal is Metal-binding term, BuryP is Burried Polar groups’ penalty, RotB is Penalty for rotatable bonds that have been frozen, and Site is active site polar interactions.

## 4. Conclusions

In the present study, the BBB permeabilities of rhynchophylline (**1**), isorhynchophylline (**2**), corynoxeine (**3**), isocorynoxeine (**4**), hirsutine (**5**) and hirsuteine (**6**), six indole alkaloids from URCU, were predicted by the MDCK-pHaMDR cell monolayer model in vitro. The results indicated that **2** and **4**–**6** had high permeability across BBB, mainly by passive diffusion, while **1** and **3** were moderately absorbed compounds with *P*-gp mediated efflux involved in their transport. The interactions of **1** and **3** with *P*-gp were further confirmed by molecule docking. In addition, analysis of the relationship between the log P, PSA and the determined *P*_app_ value of different kinds of alkaloids may help to enrich the predictive database of drug ADME/T. These new findings provide useful information for the CNS studies of URCU. Further in vivo studies should be carried out to elucidate their brain distributions.

## Figures and Tables

**Table 1:** Rh123 transport in the presence and absence of verapamil in MDCK-pHaMDR cell monolayer.

Analytes	*P*_app AP→BL_ (×10^−7^ cm/s)	*P*_app BL→AP_ (×10^−7^ cm/s)	Efflux Ratio
Rh123	8.43 ± 1.95	57.99 ± 2.59	6.88
Rh123 + verapamil	12.70 ± 2.13 *	17.07 ± 0.99 **	1.34

*P*_app AP→BL_: transport from apical (AP) to basolateral (BL) side; *P*_app BL→AP_: transport from BL to AP side. Efflux Ratio: *P*_app BL→AP_/*P*_app AP→BL_. Rh123 at 10 μM was transport with 100 μM of verapamil with an incubation time of up to 90 min. Data are the mean ± SD (*n* = 6). * *p* < 0.05, ** *p* < 0.001 vs. Rh123 group).

**Table 2:** The standard calibration curves of alkaloids **1**–**6**.

Analytes	Regression Equation	*r*^2^	Linear Range
**1**	*y* = 0.1327*x* − 0.3365	0.9979	1–150 µM
**2**	*y* = 0.1301*x* − 0.1076	0.9980	1–150 µM
**3**	*y* = 0.1109*x* + 0.0141	0.9989	1–150 µM
**4**	*y* = 0.1235*x* + 0.1916	0.9993	1–150 µM
**5**	*y* = 0.2634*x* − 0.0056	0.9997	1–150 µM
**6**	*y* = 0.1965*x* − 0.2061	0.9961	1–150 µM

**Table 3:** HPLC methodological evaluation data of alkaloids **1**–**6**.

Analytes	Concentration (μM)	Precision (%)		Accuracy (%)	Recovery (%)	Stability (%)
		Intraday	Interday			
**1**	5	0.19	6.29	107.11 ± 6.23	103.65 ± 6.59	96.32 ± 3.30
	60	0.56	6.41	100.94 ± 5.99	93.19 ± 5.70	96.86 ± 4.64
	120	0.11	4.84	104.81 ± 5.06	99.78 ± 4.89	102.59 ± 3.38
**2**	5	0.40	10.34	111.75 ± 3.75	100.83 ± 3.23	108.52 ± 1.08
	60	0.08	6.53	101.71 ± 6.59	94.44 ± 6.13	100.08 ± 2.26
	120	0.25	4.28	103.89 ± 4.32	95.94 ± 3.40	105.21 ± 3.28
**3**	5	0.57	7.33	113.34 ± 5.49	108.14 ± 5.22	106.67 ± 5.82
	60	0.07	6.66	105.42 ± 7.03	99.04 ± 6.60	102.37 ± 1.65
	120	0.44	5.75	105.18 ± 6.05	101.37 ± 5.83	101.52 ± 2.26
**4**	5	0.15	5.40	106.75 ± 2.56	110.07 ± 4.54	107.15 ± 5.06
	60	0.26	5.70	104.25 ± 3.15	95.09 ± 2.14	102.72 ± 1.97
	120	0.42	5.58	102.26 ± 2.55	95.88 ± 1.50	102.02 ± 2.43
**5**	5	4.63	12.43	110.3 ± 3.79	104.9 ± 3.60	106.1 ± 3.57
	60	0.21	5.68	102.2 ± 5.81	89.71 ± 5.10	101.9 ± 6.51
	120	0.25	6.40	106.5 ± 6.81	100.7 ± 6.44	100.5 ± 4.86
**6**	5	1.16	3.67	112.2 ± 1.91	112.7 ± 2.33	106.89 ± 3.01
	60	1.64	8.87	101.6 ± 8.83	93.75 ± 8.32	103.80 ± 5.92
	120	2.33	2.40	99.37 ± 2.36	96.60 ± 2.31	102.23 ± 4.19

**Table 4:** The bidirectional *P*_app_ values, efflux ratios of alkaloids **1**–**6** ^a^ and verapamil inhibitions.

Analytes	*P*_app AP→BL_^b^ (×10^−6^ cm/s)	*P*_app BL→AP_^c^ (×10^−6^ cm/s)	Efflux Ratio ^d^	MW	Log D ^e^ (pH = 7.35)
**1**	4.37 ± 0.37 ^(1)^	42.33 ± 2.48 ^(2)^	9.67	384.47	0.94
**2**	17.73 ± 1.20	27.73 ± 0.93	1.56	384.47	0.94
**3**	1.21 ± 0.14 ^(3)^	49.67 ± 4.71 ^(4)^	40.94	382.45	1.05
**4**	11.10 ± 1.49	20.66 ± 1.03	1.86	382.45	1.05
**5**	15.06 ± 1.32	14.11 ± 0.73	0.94	368.47	1.59
**6**	14.87 ± 1.83	11.35 ± 1.80	0.76	366.45	1.61
**1** + verapamil	22.24 ± 6.41 *	18.13 ± 1.09 ^▲^	0.82		
**3** + verapamil	17.25 ± 2.45 ^♦^	17.54 ± 0.82 ^△^	1.02		

^a^ The alkaloids **1**–**6** were at 40 μM with an incubation time of up to 90 min. ^b^ Transport of assayed alkaloids from AP to BL direction. ^c^ Transport of assayed alkaloids from BL to AP direction. ^d^ The ratio of *P*_app BL→AP_ to *P*_app AP→BL_. ^e^ Log D values at pH 7.35 were calculated by Pallas 3.3.2.6 ADEM/Tox Software. Data are the mean ± SD (*n* = 6). * *p* < 0.001 vs. ^(1)^; ^▲^ *p* < 0.001 vs. ^(2)^; ^♦^ *p* <0.001 vs. ^(3)^; ^△^ *p* < 0.001 vs. ^(4)^.

**Table 5:** The bidirectional intracellular accumulation rates and total recovery rates in transport of **1**–**6** on the MDCK-pHaMDR cell monolayer. ^a^

Analytes	AP→BL		BL→AP	
	Intracellular Accumulation (%)	Recovery Rate (%)	Intracellular Accumulation (%)	Recovery Rate (%)
**1**	0.36 ± 0.05	99.91 ± 1.15	0.40 ± 0.04	102.18 ± 0.47
**2**	0.24 ± 0.04	94.32 ± 1.43	0.37 ± 0.06	100.84 ± 1.89
**3**	n.d. ^b^	96.36 ± 1.17	n.d. ^b^	99.60 ± 1.57
**4**	n.d. ^b^	92.96 ± 1.40	n.d. ^b^	98.47 ± 0.82
**5**	16.82 ± 0.32	89.29 ± 6.15	6.19 ± 0.73	104.3 ± 1.94
**6**	19.27 ± 0.52	88.89 ± 5.99	7.52 ± 0.43	104.0 ± 1.17

^a^ Alkaloids **1**–**6** were at 40 μM with an incubation time of up to 90 min. Data are the mean ± SD (*n* = 6). ^b^ n.d.: detected less than the limit of quantification (LOQ).

**Table 6:** The log P, PSA and calculated log BB values of compounds **1**–**6**.

Compounds	1	2	3	4	5	6
log P	2.18	2.18	1.73	1.73	3.22	2.62
PSA	67.87	67.87	67.87	67.87	54.56	54.56
log BB	−0.53	−0.53	−0.60	−0.60	−0.18	−0.27
